# Digging into the extremes: a useful approach for the analysis of rare variants with continuous traits?

**DOI:** 10.1186/1753-6561-5-S9-S105

**Published:** 2011-11-29

**Authors:** Claudia Lamina

**Affiliations:** 1 Division of Genetic Epidemiology, Department of Medical Genetics, Molecular, and Clinical Pharmacology, Innsbruck Medical University, Schöpfstrasse 41, 6020 Innsbruck, Austria

## Abstract

The common disease/rare variant hypothesis predicts that rare variants with large effects will have a strong impact on corresponding phenotypes. Therefore it is assumed that rare functional variants are enriched in the extremes of the phenotype distribution. In this analysis of the Genetic Analysis Workshop 17 data set, my aim is to detect genes with rare variants that are associated with quantitative traits using two general approaches: analyzing the association with the complete distribution of values by means of linear regression and using statistical tests based on the tails of the distribution (bottom 10% of values versus top 10%). Three methods are used for this extreme phenotype approach: Fisher’s exact test, weighted-sum method, and beta method. Rare variants were collapsed on the gene level. Linear regression including all values provided the highest power to detect rare variants. Of the three methods used in the extreme phenotype approach, the beta method performed best. Furthermore, the sample size was enriched in this approach by adding additional samples with extreme phenotype values. Doubling the sample size using this approach, which corresponds to only 40% of sample size of the original continuous trait, yielded a comparable or even higher power than linear regression. If samples are selected primarily for sequencing, enriching the analysis by gathering a greater proportion of individuals with extreme values in the phenotype of interest rather than in the general population leads to a higher power to detect rare variants compared to analyzing a population-based sample with equivalent sample size.

## Background

Genome-wide association studies effectively identify new common loci and pathways, but a large amount of estimated heritability is still unexplained [1]. It is conceivable that the effects of rare variants explain some proportion of this missing heritability [2-4], because rare variants more likely reflect causative variants than common variants do. Conventional genome-wide association studies, however, are not designed to capture rare variants. The most common approach of studies aiming to identify rare variants is based on the assumption that rare variants with large effects are enriched in the extremes of the phenotype distribution [3,5].

If resources are limited, sequencing the extreme tails of a phenotype distribution in the search for rare variants has been successful, for example, in studies on candidate genes for lipoprotein metabolism [5-7] or type 1 diabetes [8]. Is it a failure to neglect the “uninformative middle” of the distribution if there is no monetary pressure to do so? When applying this extreme trait approach to a continuous phenotype, much of the overall distribution of the phenotype is neglected and some rare genetic variants with just moderate effects may be missed. In contrast to this assumption, the extreme phenotype approach has been more successful than linear regression on all values in a recent study on free fatty acids [9]. In this study, a linear regression on the complete phenotype distribution was used and methods designed for rare variants in a case-control design [10] by focusing on the extremes of the phenotype. An excess of rare variants in the upper tail of the distribution is shown by the case-control design.

In light of these recent findings and the expected role of rare variants, is linear regression still the gold standard? It might be conceivable that an excess of rare variants in the extremes would be better detected by choosing good discriminative cut points for the extreme values using appropriate statistical methods. On the other hand, a lot of information might be lost, leading to diminished power. Is this power loss merely due to a smaller sample size?

With these considerations in mind, my aim in this analysis of the Genetic Analysis Workshop 17 (GAW17) data set is to detect genes with rare variants that are associated with quantitative traits using two general approaches: analyzing the association with the complete distribution of values by means of linear regression and performing statistical tests based on the tails of the distribution.

## Methods

### Data

In this analysis, I use data prepared for GAW17. These data include 697 unrelated individuals from the 1000 Genomes Project originating from 6 populations (African, Asian, European) [11]. The genotypes were held fixed, and three normally distributed phenotypes (Q1, Q2, and Q4) were simulated in 200 replication data sets. In this analysis, I use all 200 replicates on all three continuous traits. The genotype data are composed of 21,356 single-nucleotide polymorphisms (SNPs) clustered in 3,205 genes, 2,874 of which also include rare variants. The quantitative trait Q1 has been simulated to be influenced by 39 SNPs in 9 genes, and Q2 by 72 SNPs in 13 genes; Q4 is not influenced at all by genes in this data set.

### Collapsing rare variants within a gene

Because of the nature of rare variants, analyzing each of them individually lacks adequate power. Several kinds of collapsing methods for rare variants have been proposed. The primary idea is to group rare variants together in such a way that individuals with at least one rare variant are tested against others who do not have any rare variant [12,13]. In this analysis, I group rare variants on the gene level, indicating only whether each individual has at least one rare variant within one gene or not, corresponding to a dominant allele coding. For each gene, I created one indicator variable, *I*(number of rare variants within the gene > 0) (= *I*(rare)). I define rare variants as variants with a minor allele frequency (MAF) less than 5%.

### Analysis using all values of the continuous traits

For the association analysis of the rare variants with all values of the continuous traits, I use linear regression models and collapsed rare variants into one indicator variable. Morris and Zeggini basically proposed this idea in the combined multivariate and collapsing (CMC) method [14]. The expectation of the phenotype *Y* is modeled as follows for each gene with rare variants:(1)

where *n* is the number of individuals, *α* the intercept and and *β*_rare_ is the expected increase of the phenotypic trait in individuals with at least one rare variant within the respective genes compared to individuals without any rare variant. To evaluate possible population stratification issues, I performed a population-stratified analysis by running the linear regression separately in three homogeneous groups (Africans, Asians, Europeans) followed by a consecutive random effects meta-analysis.

### Analysis based on the extreme tails of the distribution

The extreme tails are defined by values smaller than 10% quantiles versus values greater than 90% quantiles (i.e., the bottom 10% and top 10% of the categorized trait). In addition, I created a dichotomized trait (the bottom 90% versus the top 10%). In both cases, each gene can be presented in a 2 × 2 table. Therefore we can apply methods that have been proposed for case-control studies only. I use the following methods and compare them in this analysis:

1. Fisher’s exact test on each of the 2 × 2 tables corresponding to the cohort allelic sum test (CAST) method proposed by Morgenthaler and Thilly [13].

2. Beta test proposed by Li et al. [10], which compares proportion differences of rare variants between affected and unaffected subjects. The test does not involve any distribution approximation and can therefore be used as an exact test for the analysis of 2 × 2 tables.

3. Weighted-sum (WS) method proposed by Madsen and Browning [15], which groups rare variants and scores each individual using a weighted sum of rare variant counts. The WS method is a test for an excess of rare variants in affected individuals.

More details on the collapsing methods used in this analysis (CMC, CAST, WS) can be found in Dering et al. [16].

Because the analyses on the categorized trait are based on only 20% of the sample size of the continuous trait, I evaluate the effect of sample size on the categorized trait using the following approaches:

1. Enriching the tails: In the analysis of each of the first 100 replicates, additional replicates were sampled from replicates 101–200 by chance to increase the sample size of the top and bottom 10%. This enrichment was performed by adding one or two additional data sets, leading to 40% or 60%, respectively, of the original sample size of the continuous trait.

2. Widening the tails: The sample sizes were increased by shifting the limits of the categorized trait Q1 closer to the median: The top and bottom 20% were included to attain 40% of the original sample size, and the top and bottom 30% were included to reach 60% of the original sample size.

## Results

### Comparing type I error and power

For Q4, which serves as a control phenotype, the type I error was inflated for the linear regression (0.10) and pretty much preserved to slightly elevated for the other methods (0.02–0.07). For Q2 and especially Q1, the type I error was inflated, with a maximum of 0.16 for the linear regression in Q1. The WS and beta methods yielded similar rates throughout (using the categorized trait in Q1, 0.09 for WS, 0.11 for beta; in Q2, 0.04 for WS, 0.05 for beta). The population-stratified analysis eliminated the inflation in Q4 (0.04) and diminished it for the other traits (0.11 for Q1 and 0.06 for Q2).

**Figure 1:**
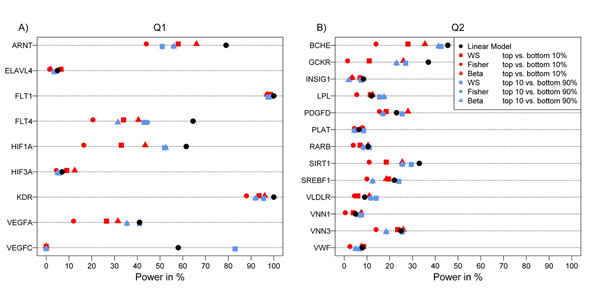
**Power to detect genes with rare causal SNPs using different methods.** Showing the power to detect (A) each of the 9 genes including causal SNPs for Q1 and (B) each of the 13 genes for Q2 for all methods used: linear regression (black dots); weighted sum (WS), Fisher’s exact test, and beta test on the categorized trait (top 10% versus bottom 10%) (red dots); and all three methods on the dichotomized trait (top 10% versus bottom 90%) (blue dots). Power was calculated as the percentage of nominally significant *p*-values using all 200 replicates.

Figure 1 shows the power to detect the true associated genes for Q1 and Q2 based on a nominal significance level of 0.05. Power ranges from 97.5% to 100% for *FLT1* and from 1.5% to 6.5% for *ELAVL4*; both of these genes are similarly likely to be detected by all methods. For other genes, there is a high variation in power with regard to method. Overall, linear regression is the most powerful method for almost all genes and both traits, followed by the methods using the dichotomized trait. Comparing the three methods used for the categorized trait, the beta method shows the highest power for almost all genes, followed by the WS method. For Q2, power is generally lower (maximum 45.5% for *BCHE*), but the overall pattern remains the same. Correction for multiple testing using a simple Bonferroni correction would have detected only *FLT1* using linear regression on Q1 with adequate power (91%).

### Effect of sample size

I used sample-size-enriched data sets to evaluate the effect of sample size on the extreme phenotype approach compared to the gold standard of linear regression. Because the beta method performed the best of all methods in the extreme phenotype approach and also outperformed the WS method with regard to computing time, I used only the beta method for this analysis step.

**Figure 2:**
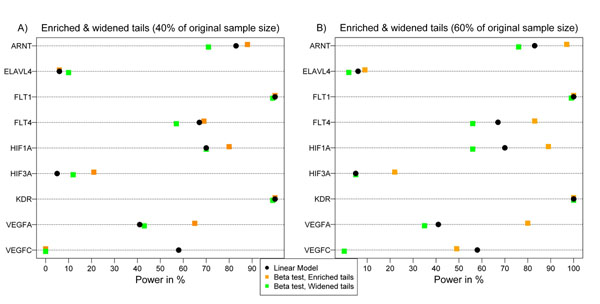
**Increasing the sample size by enriching or widening the tails and effect on power**. The power to detect each of the nine genes including causal SNPs for Q1 using linear regression and the beta test with increased sample size. Orange dots refer to the analyses enriched by adding further individuals with extreme trait values: (A) One additional replicate was added to gain 40% of the original sample size; (B) two additional data sets were added to gain 60% of the original sample size. Green dots represent the analyses based on the widened tails: (A) top 20% versus bottom 20%; (B) top 30% versus bottom 30%. Power was calculated as the percentage of nominally significant *p*-values using the first 100 replicates.

**Figure 3:**
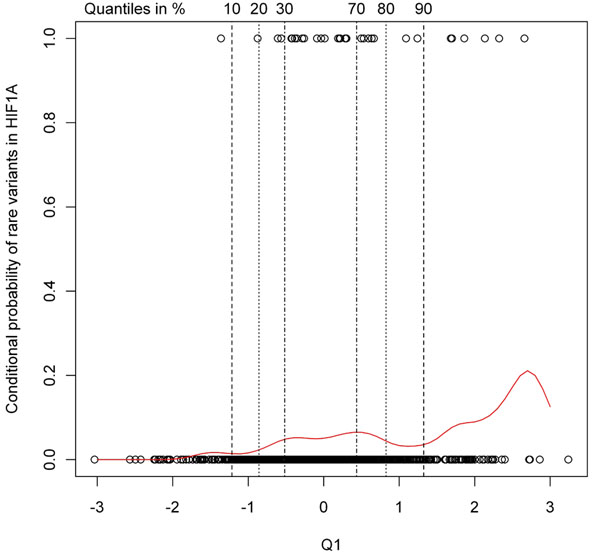
**Conditional density plot: probability of carrying a rare variant conditional on Q1 values**. The dots at the upper or lower border of the plot depict the values of Q1 (given on the *x*-axis) for each individual with rare variants (upper dots) or without rare variants (lower dots). Based on the observed distribution, the curve gives the probability of carrying a rare variant conditional on Q1 values. The position of the upper and lower 10%, 20%, and 30% quantiles of Q1 are marked by vertical lines.

Figure 2 shows both the enriched-tail and the widened-tail approaches. For each added data set (enriched method), power increases, whereas widening the tails to include individuals with phenotypes closer to the median decreases the power. Doubling the sample size in the extreme phenotype approach (top 10% versus bottom 10%) yields a comparable or even higher power than linear regression, with the exception of the *VEGFC* gene in the association analysis of Q1. For clarification of this behavior, a conditional density plot is shown in Figure 3. In this figure the distribution of the phenotype conditional on the rare variants is exemplified on *HIF1A* using the first replicate. The curve represents the probability of carrying at least one rare variant in the *HIF1A* gene conditional on Q1 values, which is highest for values above the 90th quantile with as low as 0 probability for values below the 10th quantile. Moving the cut points to the median also enlarges the sample size, but the information added is rather noninformative and thus the power decreases.

## Discussion

I applied two different general approaches to the analysis of rare variants in the GAW17 data set: using all values of the continuous trait distribution in a linear regression and comparing the proportion of rare variants in the upper tail of the distribution (upper 10%) versus the rest (lower 90%) or in the lower tail (lower 10%) using methods reported for the detection of rare variants in case-control studies.

Inflated type I errors is an issue in the analysis of the GAW17 data set. Consistent false-positive findings have been reported over different methods and all replicates [17]; these false positives are partly due to long-range linkage disequilibrium between genes. The higher power of the linear regression is gained by a higher type I error rate, which is a natural consequence but should be kept in mind. Appropriately adjusting for population stratification could reduce the inflation, though. Similar type I error rates between the WS method and the beta method indicate that neither of these methods can be preferred over the other in this regard. Because genetic effects were simulated in GAW17 using a normal distribution, it was expected that linear regression would outperform the other extreme phenotype approaches with regard to power.

The WS and beta approaches come down to a 2 × 2 table for which well-known methods could also be applied. From the three methods applied, the beta test and the WS method are the most powerful, indicating that methods that have been primarily developed for the detection of rare variants should be preferred over a simple but overconservative Fisher’s exact test.

Another question handled in this analysis was whether the higher power of linear regression was simply due to higher sample size. Enriching the tails by adding individuals with extreme phenotypes led to an increase in power, but widening the tails led to a decrease. Therefore, it is not the size that matters but the selection of samples. It should be noted, however, that the enrichment method artificially added “genotypic twins” because genotypes were held fixed in the simulated replicates. This would not happen in the real world, and it is not clear to what extent this twinning affected the results.

In this analysis a sample size that is 40% as high as the original sample size is sufficient to gain at least the same power as found with linear regression. This result is in line with the findings of Yang et al. [18], who compared a quantitative trait analysis with a case-control design assuming a threshold model of disease. Yang and colleagues showed that for diseases with a prevalence smaller than 10%, a balanced case-control study needs a smaller sample size than a quantitative trait analysis does to gain equivalent power. Such a threshold model for a rather rare disease can easily be transferred to the underlying idea of an extreme phenotype approach of a continuous trait.

If such an approach is chosen, the question remains, How should extreme values be defined? In this analysis, I chose the cut point of 10% as a trade-off between really extreme values and sample size. Conditional density plots for many genes and replicates indicated that using the bottom and top 10% of the distribution provided a good discrimination between individuals with at least one or no rare variant without losing too much data and therefore information. In general, this decision has to be based on the studied phenotype and the sample size, which is available or can be sequenced.

## Conclusions

What do these findings mean for sample selection and analysis strategy? If a complete population-based study is available for sequencing, then the statistical analysis should not focus on the extremes alone because a standard linear regression including all values is expected to provide the highest power to detect rare variants. Extreme phenotype approaches, however, should be conducted in addition, because this approach has been successful in real study situations. If samples are selected primarily for a sequencing approach, then enriching the analysis by gathering a greater proportion of individuals with extreme values in the phenotype of interest than in the general population leads to a higher power to detect rare variants compared to analyzing a population-based sample with equivalent sample size. With such a selection strategy using rare-variant-approved methods, such as the beta or WS test, money on sequencing can be saved or spent to include additional samples. However, these collapsing methods can only point to genes or sets of SNPs; detecting the truly functional variants is still necessary.

## Competing interests

The author declares that there are no competing interests.

## Author’s contributions

CL developed the design of the study, performed the statistical analysis and wrote the manuscript.
